# Fully Synthetic Non‐Carbohydrate Heparin Mimetics—Perspectives for Therapeutic Anticoagulation and Beyond?

**DOI:** 10.1002/ardp.70239

**Published:** 2026-04-17

**Authors:** Katrin Nekipelov, Vito Ferro, Gerd Bendas

**Affiliations:** ^1^ Pharmaceutical Department University of Bonn Bonn Germany; ^2^ School of Chemistry and Molecular Bioscience University of Queensland Brisbane Australia

**Keywords:** anticoagulation, Heparin, Heparin mimetics, synthetic polymers

## Abstract

Heparin is one of the oldest drugs on the market. Although clinically well established as an anticoagulant for decades, owing to its natural origin and heterogeneous glycosaminoglycan polysaccharide structure, ensuring consistent quality and reproducible biological activity remains challenging. While these facts argue against future applications of heparin in clinical routine, an increasing number of studies reveal additional potential therapeutic applications of heparin beyond its anticoagulant efficacy, that is, as an antiviral strategy or as a multi‐targeted approach in oncology. Therefore, heparin appears to be a structural template for pleiotropic compounds. To combine the beneficial pleiotropic properties of heparin with improved reproducibility and safety, fully synthetic heparin mimetic substances have increasingly become a focus of scientific research. These studies enable more detailed structure–activity relationships of heparin in its interaction with proteins or diseases to be elucidated, thereby facilitating the development of novel compounds for potential therapeutic use. This review focuses in particular on fully synthetic non‐carbohydrate heparin mimetic polymers with anticoagulant activities, as well as their properties beyond anticoagulation. Advances in the development of such mimetics, especially over the past decade, allow increasingly robust conclusions to be drawn regarding their efficacy and contribute to a deeper understanding of this class of compounds.

## Graphical abstract

Fully synthetic sulfated non‐carbohydrate polymers for structurally mimicking the polysaccharide glycosaminoglycan heparin in its anticoagulant activities with further prospects to pleiotropic effects in oncology and in antiviral approaches.

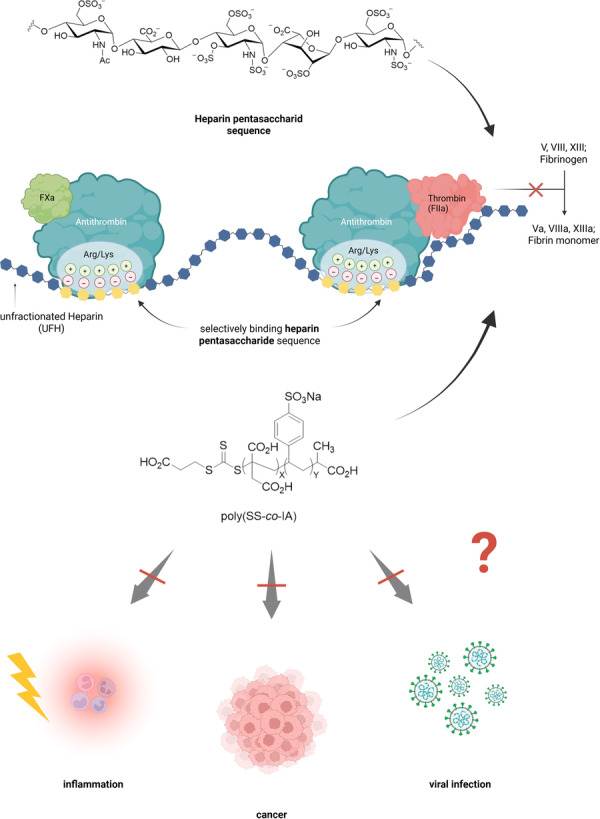

## 1 Introduction

### 1.1 HEPARIN—Structure and a Historic View on Its Discovery

Heparin was the first drug used for rational anticoagulation in medicine for therapeutic purposes. It was initially discovered in 1916 by Jay McLean, who identified it by chance while conducting research on thromboplastic substances. At that time, the substance was believed to be a phosphatide and was therefore termed “heparphosphatide.” Surprisingly, unlike the cephalin expected to study, it displayed anticoagulant rather than procoagulant activities [1].

In 1933, Charles and Scott succeeded in the purification of heparin from bovine liver, and by 1937, it was administered therapeutically to humans for the first time [2]. Since then, heparin has played a pivotal role in anticoagulant therapy. Heparin occurs naturally in various organs of higher animals, including the liver, lungs, intestinal tissues, and blood. For therapeutic use, however, it is primarily extracted from porcine intestinal mucosa and subsequently converted into its sodium or calcium salt through enzymatic or chemical treatment [3]. Heparin is a linear polymer, which consists of alternating units of d‐glucosamine and uronic acid, where the uronic acid residues consists of 75%–95% l‐iduronic acid (l‐IdoA) and 5%–25% of d‐glucuronic acid (d‐GlcA), depending on the origin (animal species and organ) [4, 5, 6]. Owing to its extensive sulfation and the presence of carboxyl functionalities, heparin represents one of the most highly negatively charged biological molecules, with an average of approximately 2.7 sulfate groups per disaccharide unit. The trisulfated disaccharide is the most common motif, which accounts for the uneven number. Nevertheless, the exact structural composition and distribution of disaccharides remain incompletely defined due to the micro‐heterogeneity of heparin. This heterogeneity arises from diverse substitution patterns within the constituent monosaccharides. In glucosamine residues, the amino group may be acetylated, sulfated, or unsubstituted. Furthermore, the OH‐groups at C‐3 and C‐6 positions of glucosamine can either be O‐sulfated or unsubstituted. Uronic acid residues also show considerable variability, not only in their relative abundance but also through modifications such as the presence or absence of 2‐O‐sulfation [4, 6, 7, 8, 9]. The average chain length of unfractionated heparin (UFH) is 10–20 kDa, at the most frequent 15 kDa (approximately 45 monosaccharides) but varies depending on the batch and manufacturer [7]. In 1976, Rosenberg, Lindahl, and Barrowcliffe independently demonstrated that only one‐third of heparin molecules exhibit high affinity for antithrombin III, providing the first evidence for a distinct active binding site within the heparin chain. Subsequent structural analyses by the groups of Lindahl and Casu led to the precise elucidation of the critical pentasaccaride sequence, which was later successfully synthesized by Maurice Petitou in 1983 at the Choay laboratory in France [10, 11, 12, 13].

**Figure 1:**
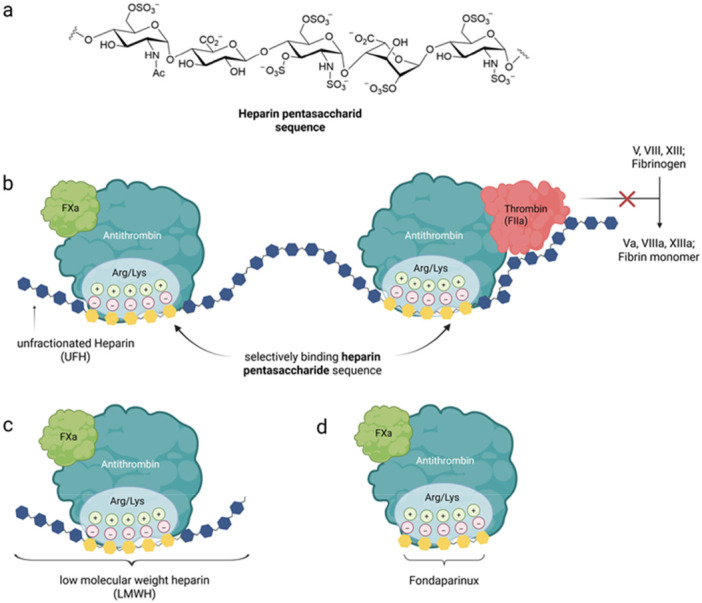
Schematic representation of the structure and the mechanism of action of heparin derivatives. (a) Heparin pentasaccharide as the minimal antithrombin binding sequence. (b) Mechanism of action of unfractionated heparin. Binding to antithrombin, heparin induces a conformational change, triggering an almost thousand‐fold higher affinity to form a ternary complex with thrombin and factor Xa, blocking the indicated coagulation factors with strong downstream consequences to suppress the coagulation cascade. (c) Mechanism of action of LMWHs. The shortened chain length of these heparin derivatives still exhibits strong antithrombin binding and inhibition of factor Xa, but without forming a ternary complex with thrombin. (d) Mechanism of action of fondaparinux. The completely synthetic pentasaccharide of heparin represents the active binding sequence to antithrombin. The high negative charge density of the pentasaccharide enables ionic interactions with the positively charged heparin‐binding domain, mainly mediated by arginine and lysine residues. It acts primarily as an indirect, highly selective factor Xa inhibitor.

The pentasaccharide (Figure 1a) comprises two repeating disaccharide units: N‐acetylated glycosamine (d‐GlcNAc) with d‐GlcA, and N‐sulfated glucosamine (d‐GlcNS) with l‐IdoA—arranged around a central glucosamine residue bearing a critical 3‐O‐sulfation. The defined sequence is α‐d‐GlcNAc,6S‐β‐d‐GlcA‐α‐d‐GlcNS,3,6S‐α‐l‐IdoA2S‐α‐d‐GlcNS,6S. This highly specific arrangement, stabilized by 1→4 glycosidic linkages, constitutes the minimal antithrombin III binding site and underlies the potent anticoagulant activity of heparin [14, 15, 16]. Based on these insights, extensive efforts over the subsequent two decades were directed toward providing the fully synthetic active pentasaccharide as a drug. In 2002, fondaparinux (Figure 1d) obtained an approval as the first fully synthetic anticoagulant representing a heparin analog principle. Notably, the first sugar unit of the pentasaccharide in fondaparinux is N‐sulfated instead of N‐acetylated as predominantly found in porcine heparin. Although the N‐sulfated variant also occurs in porcine heparin in minor amounts, it represents a significantly smaller fraction while displaying approximately twofold higher anti‐Xa activity [17].

### 1.2 HEPARIN—Mode of Action and Therapeutic Drawbacks

Through allosteric binding of heparin to the serpin antithrombin III, a conformational change is induced within the protein, which subsequently leads to its activation. This conformational transition of antithrombin by binding the indicated pentasaccharide is primarily responsible for the accelerated inhibition of coagulation factor Xa, whereas thrombin binding to antithrombin III is dependent on a minimal critical chain length of heparin of 18 saccharide units (Figure 1b) [18].

**Figure 2:**
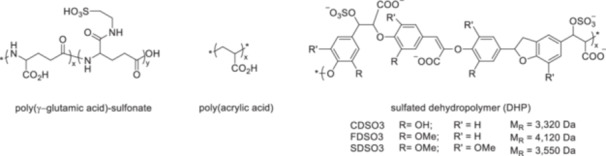
Synthetic polymers as heparin mimetics. Abbreviations: CDSO3, sulfated dehydropolymer of caffeic acid; FDSO3, sulfated dehydropolymer of ferulic acid; SDSO3, sulfated dehydropolymer of sinapic acid.

Thus, concurrent binding of thrombin and antithrombin promotes the formation of the ternary bridging complex [19, 20]. The dosage of heparin is determined based on its pharmacological activity rather than its mass [21]. To achieve a more predictable anticoagulant effect with longer half‐life and improved bioavailability compared with UFH, low molecular weight heparins (LMWHs) have become established in clinical practice since the 1980s. They are produced by chemical or enzymatic depolymerization of UFH and consist of shorter polysaccharide chains with an average molecular weight of approximately 4–5 kDa. Owing to their reduced chain length, LMWHs exhibit less thrombin (factor IIa) inhibition relative to UFH, while maintaining effective factor Xa inhibition (Figure 1c). In addition, their subcutaneous administration option makes them attractive for outpatient use [22].

Due to its natural extraction, heparin exhibits considerable variability in molecular weight and anticoagulant activity. Moreover, pandemics or other animal diseases pose a significant risk of quality deterioration or even contamination. A striking example is the 2008 heparin crisis, when the commercial drug was adulterated with oversulfated chondroitin sulfate, resulting in 80–150 documented deaths and more than 350 adverse events in the United States alone [23, 24]. The direct dependency on pigs as the primary source of heparin and their susceptibility to disease were further highlighted during the African swine fever outbreak of 2018. The massive loss of pig herds in China directly correlated with global shortage of heparin raw material [25, 26].

In addition to challenges related to its extraction, heparin presents a significant clinical risk due to its strong negative charge: heparin‐induced thrombocytopenia (HIT). Type I HIT typically appears within the first 3 days of therapy, is transient, and usually causes only mild symptoms. In contrast, Type II HIT develops with a delay of 4–10 days, is antibody‐mediated, and can result in severe complications with mortality rates of up to 30%. Although relatively rare, this immunological reaction represents one of the most serious adverse effects of heparin therapy [27, 28]. This side effect is partly driven by the increased sensitivity of platelets through the interaction of the surface integrin αIIbβ3. The resulting conformation change promotes platelet activation and the release of platelet factor 4 (PF4) from the α‐granula of platelets. PF4 is a positively charged chemokine that binds strongly to the negatively charged heparin molecules, with the strength of interaction depending on the heparin chain length. The resulting heparin‐PF4 complexes are recognized by IgG antibodies, which are linked to thrombotic events. These immune complexes (heparin‐PF4‐IgG) activate platelets via FcγIIa receptors, ultimately triggering platelet aggregation and thrombocytopenia [29, 30]. LMWHs are associated with a lower incidence of HIT, while the pentasaccharide fondaparinux has not been shown to trigger this reaction [31].

Due to heparin's negative charge, there is a potential for interaction with other proteins. Cationic proteins such as protamine are used as an antidote to neutralize this negative charge. Recent studies aimed at improving the safety of heparin have identified positively charged guanidine‐containing heparin mimetics that do not have anticoagulant effects and which can antagonize the action of heparin while simultaneously demonstrating direct carbohydrate–carbohydrate interactions [32].

Due to these complications and its relatively short half‐life of 60–90 min after i.v. administration, UFH is being phased out of current guidelines [33]. LMWH or direct oral anticoagulants (DOACs) are now preferred over UFH for the treatment of conditions such as venous and arterial thromboembolism or pulmonary embolism.

In light of the structural heterogeneity, the indirect mode of action and the animal source with potential problems in the supply chain, heparin, even LMWHs appear old‐fashioned drugs in times of rational drug design. Nevertheless, heparin has not been superseded since pleiotropic effects are driving renewed interest in other therapeutic applications, often beyond anticoagulation. For example, several studies have demonstrated potential anti‐cancer activities of UFH and LMWH. These effects were attributed to their ability to prevent cancer cells from adhering to the vascular endothelium in terms of their haematogenous metastatic route and to bind tumor‐derived proangiogenic factors essential for angiogenesis [34, 35, 36]. Owing to its size and charge density, the heparin molecule can interact with cell adhesion molecules or different growth factors or chemokines, thereby suppressing tumor growth, metastasis, and invasion [37]. Furthermore, early studies in the 1980s showed that heparin inhibits the heparanase‐mediated degradation of heparan sulfates in the extracellular matrix, both in active cells and in isolated heparanase. Referring to these findings, further heparanase inhibitors have been designed using heparin as a structural scaffold. Several heparin‐derived inhibitors have been tested in initial clinical trials for oncological treatment [38]. Beyond its direct clinical relevance, heparin has also provided important insights into the signaling pathways of heparan sulfate proteoglycans (HSPGs). For example, it has helped to elucidate the mechanistic role of syndecan‐1 in tumor development as well as the thrombin‐driven activation of platelets by tumor cells, a process that facilitates metastatic spread [39, 40].

To further differentiate whether the pleiotropic effects of heparin in oncology only result from anticoagulation, a comparative view on DOACs is essential. Although DOACs have demonstrated effects beyond their anticoagulant activity in initial clinical and preclinical studies, these pleiotropic effects appear to be less pronounced than those reported for heparin. DOACs have been shown to exert anti‐inflammatory, antifibrotic, and antioxidant properties. However, these effects are thought to arise from the modulation of factor Xa and thrombin, which regulate various (patho)physiological processes, including inflammation, atherothrombosis, and angiogenesis through activation of protease‐activated receptors (PARs). By inhibiting thrombin and factor Xa, DOACs can interfere with PAR‐mediated signaling pathways and thereby modulate these processes [41, 42, 43, 44].

Studies in animal models investigating the effects of DOACs on tumor growth and metastasis have shown that DOACs induced neoplastic changes in rat carcinogenicity, but showed no effect in mouse xenotransplant models [45, 46]. In contrast, results from syngeneic mouse models indicate that the impact on tumor growth and metastasis depends on both the timing of DOAC administration and the type of cancer model used [47]. Individual studies have reported potential antitumor effects. For example, the administration of the thrombin inhibitor dabigatran was shown to reduce the dissemination of breast cancer cells and to produce synergistic effects in reducing tumor growth when combined with alkylating agents such as cisplatin or cyclophosphamide. However, increased cytotoxicity of gemcitabine was observed when it was administered concomitantly with dabigatran [48, 49, 50, 51]. The oral FXa inhibitors apixaban, rivaroxaban, and edoxaban have demonstrated antitumor effects in isolated in vitro and in vivo studies. However, these effects were generally observed at doses higher than those used clinically, which were also associated with increased cytotoxicity [52, 53, 54, 55, 56]. Given the very limited data and the often contradictory findings regarding the effects of DOACs on tumor proliferation, further research is required before conclusions can be drawn about their potential antitumor properties.

Apart from its promises in tumor therapy, heparin has recently gained considerable attention as a life‐saving antiviral agent during the COVID‐19 pandemic. Studies have shown that heparin binds to the SARS‐CoV‐2 spike protein and interferes with its interaction with extracellular heparan sulfates that act as cellular co‐receptors for anchoring and for stabilizing the open conformation essential to facilitate its binding to angiotensin‐converting enzyme 2 (ACE2). In this way, heparin is considered to act as an inhibitor of viral entry to host cells. In addition to UFH, which demonstrated such inhibition of viral infectivity in vitro at concentrations comparable to those used in anticoagulant therapy [57, 58, 59], a similar mode of action has been described for pentosan polysulfate, a chemically sulfated derivative of the plant‐derived xylan pentosan [60].

These findings emphasized a novel view on an old and seemingly outdated class of drugs, offering a broader therapeutic potential beyond anticoagulation. While increasingly supplanted by the DOACs as newer anticoagulants, the polymeric principle of heparin remains a highly attractive scaffold for novel polymeric compounds with considerable promise for diverse applications.

To retain the positive properties of heparin while avoiding or reducing its disadvantages, heparin‐like polymeric structures have been developed during the last decades, either as novel anticoagulants able to overcome potential drawbacks of heparin, or novel active compounds emphasizing the pleiotropic functionalities of the original structure implicating indications beyond anticoagulation. In addition to generating probable new drugs, further insight and structural understanding of processes that have been addressed by heparin appears to be a further benefit for these approaches.

## 2 Heparin Mimetics

### 2.1 General Prospects

During the last two decades, multiple approaches have been described to simulate and transfer the structural scaffold given by heparin as a highly negatively charged macromolecule to other structural templates to obtain novel active compounds, either as anticoagulants, or to make use of the pleiotropic heparin effects and emphasize other targeted activities.

Besides the semisynthetic modification of the original heparin molecule by de‐ or oversulfation, oxidative ring opening, or other chemical derivatizations to fine‐tune certain biological effects (anti‐inflammatory, heparanase inhibition, anti‐angiogenetic, and further) [61, 62, 63], which will not be considered here, two main further strategies stand out in particular.

On the one hand, the (over)sulfation of natural, non‐carbohydrate‐based compounds has attracted much attention, mainly in the search for alternative anticoagulant drugs.

On the other hand, the controlled synthesis of heparin mimicking synthetic polymers of non‐carbohydrate nature appears to be a promising strategy. While both approaches ensure precise control of structure, purity, and biological activity, a key advantage of fully synthetic polymers is the ability to selectively reproduce specific properties of heparin by tailoring the molecular structures. This enables the development not only of anticoagulant heparin mimetics but also depending on the therapeutic goal, non‐anticoagulant antiviral agents, antiangiogenic derivatives, cytokine‐scavenging polymers, and many others. Such versatility supports the creation of highly targeted drugs and provides deeper insights into the structure–activity relationships (SARs) of heparin across different biological mechanisms. Consequently, heparin mimetics not only yield well‐defined, pure compounds but also serve as powerful tools for understanding disease mechanisms, thanks to their adaptability and structural diversity.

In this review, we focus on defined synthetic structures that replicate the therapeutic effectiveness of heparin while reducing the side effects and complications linked to its extraction and dosing. Since the first class of compounds, sulfated natural products with focus on anticoagulation has been excellently reviewed by Paluck et al. in 2016 [64], who provided a comprehensive overview of those strategies and Nahain et al. [65], who provided a detailed overview of anticoagulant heparin mimetics in 2018, we are content to update these data, but mainly focus the review on the synthetic heparin mimicking polymers with a broader range of applications.

### 2.2 Sulfated Natural, Non‐Carbohydrate Based Compounds as Potential Anticoagulants

In the pursuit of structurally well‐defined anticoagulant compounds, the modification of naturally occurring molecules represents one of the most elegant and efficient strategies for generating promising candidates. Flavonoids, benzofurans, coumarins, and related scaffolds have proven particularly suitable for this purpose. The aromatic, oxygen‐containing heterocycles with fused ring systems provide electron‐rich frameworks, which, up to sulfation, acquire a highly negative charge and enhanced capacity for biological interactions. Such systems have therefore emerged as valuable leads for anticoagulant drug discovery.

This section focuses on the derivatization of these natural scaffolds and the advances achieved in this area over the past decade (Table 1). Among the most influential contributors are the Desai research group, whose pioneering work has been further expanded by Al‐Horani and co‐workers through a series of impactful studies. The anticoagulant activities of these compounds were evaluated based on their ability to inhibit thrombin, factor XIa or factor XIIIa, to activate antithrombin or by assessing prolongation of clotting times in plasma assays or in the earlier stages of these investigations, HINT analyses such as binding studies on the aforementioned proteases. The focus on factors XIa and XIIIa stems from their distinctive pharmacological profile: inhibition of these enzymes is associated with reduced bleeding risk compared with conventional anticoagulant targets, offering a safer therapeutic strategy for thrombosis prevention. The focus in this context is placed specifically on the allosteric inhibition of the proteins. During the development of natural product‐based anticoagulant heparin mimetics, the emphasis gradually shifted from flavonoid derivatives (Compounds **1–5**) to isoquinoline analogs (Compounds **6–7**) and ultimately to sulfated benzofurans (Compounds **8–11**). As development progressed, further structural classes were introduced, but without any dominant clustering.

**Table 1:** Overview of sulfated natural, non‐carbohydrate heparin mimetics.

Nr	Structure	Structure class	DS	Measurement of anticoagulation	Ref
1	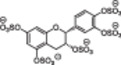	Epicatechin sulfate (+) catechin sulfate (−) catechin sulfate	5	HINT analysis: Slightly increased activation of AT – just 10 fold, but no binding in the PBS	[68]
2	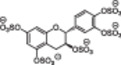				
3	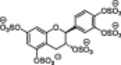 MW: 685,52 g/mol				
4	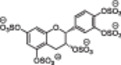 MW: 685,52 g/mol	Epicatechin sulfate	5	HINT analysis: Slightly increased activation of AT – eightfold Lead‐structure	[69]
5	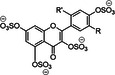 MoS: R=H R’=OSO_3_^‐^ QS: R=OSO_3_^‐^R’=H MW: 697,49 g/mol	Epicatechin sulfate Morin sulfate Quercetin sulfate	5	HINT analysis: Slightly increased activation of AT – 8 to 20 fold	[70]
6	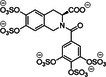 IAS_5_ MW: 755,55 g/mol	Sulfated tetrahydro‐isoquinoline‐acid /ester	4‐5	Activity was determined based on binding affinities to AT and following AT activation – a 30 fold inhibition of IAS_5_ was shown	[71]
7	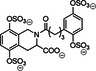 Compound 20c MW: 674,52 g/mol	Sulfated tetrahydro‐isoquinoline	4	Determination of K_D_ for the interactions with AT and the inhibition of FXa through AT activation – compound 20c achieves almost 80‐fold activation	[72]
8	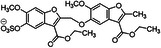	Sulfated benzofuran dimer	1	APTT and PT tests demonstrate anticoagulant properties, but significantly weaker (100 to 250‐fold) compared to oligomeric sulfated LMWLs	[73]
9	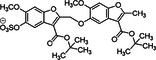 Compound 12a & 12c MW: 12a = 577,53 g/mol MW: 12c = 633,64 g/mol				
10	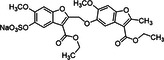 Compound 12a MW: 600,52 g/mol	Sulfated benzofuran dimer	1	Pure SAR analysis: substance identified ARG173 in exosite 2 of thrombin as critical allosteric binding site – Lead structure for further allosteric anticoagulants	[74]
11	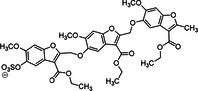 Compound 9a MW: 825,77 g/mol	Sulfated benzofuran trimer	1	APTT & PT: 10‐fold more potent than the first generation dimers but 10‐fold less potent than polymeric sulfated LMWLs	[75]
12	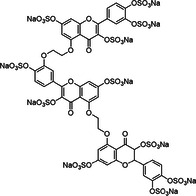 Compound 13 MW: 2083 g/mol	Undecasulfated trimeric flavone	11	High factor allosteric and reversible inhibition of FXIIIa‐mediated fibrin cross‐linking, thereby reducing clot stabilization and up to 26 fold selectivity over other proteases (FXa, thrombin, papain)	[76]
13	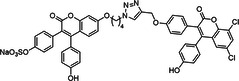 Compound 3g MW: 982,77 g/mol	Sulfated coumarin dimer	1	Direct thrombin activity assay: Thrombin is inhibited allosterically and with moderate potency via exosite 2, with high selectivity – intends to reduce potency in order to minimize side effects	[77]
14	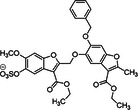 Compound 2c MW: 653,63 g/mol	Sulfated benzofuran dimer	1	Chromogenic substrate hydrolysis assay: Sub‐maximal allosteric inhibition of thrombin by a small synthetic molecule – intends to reduce potency in order to minimize side effects	[78]
15	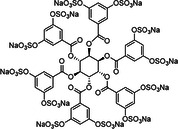 MW: 2221,26 g/mol	Sulfated chiro‐inositol	13	Very potent and selective inhibition of FXIa in chromogen substrate assay, dose dependent APTT prolongation without PT prolongation, TEG and HAS showed reduced clot stability, *in vivo* test demonstrated reduced thrombus formation without significant increase in bleeding. Reversible effect with antidote treatment	[79]
16	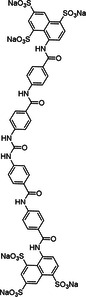 Compound 16 MW: 1401,10 g/mol	Diphenyl urea derivative	6	Allosteric, selective inhibition of FXIa without relevant toxicity APTT/PT coagulation tests – increasing clotting time Further SAR findings: High sulfation tends to be beneficial for anticoagulation, but too much sulfation results in inactivity; Longer linkers and para‐substitution improve protein binding	[66]
17	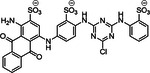 Compound 13 MW: 771,12 g/mol	Sulfated triazine derivative (13)	3	Allosteric, selective FXIIIa inhibitors, with compound 16 being even more selective APTT & PT – increase in clotting time Both without significant cytotoxicity	[80]
18	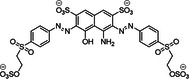 Compound 16 MW: 899,83 g/mol	Azo‐naphthalene derivative (16)	4		
19	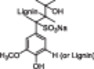 MW: > 354,39 g/mol	Non‐acetylated lignosulfate (NALS) polymer	> 1	Potent selective FXIa inhibitor with low toxicity; APTT & PT tests – mainly prolongs APTT; Economically attractive, as it is produced as a byproduct in lignin production	[81]

Abbreviations: APTT, activated partial thromboplastin time; AT, antithrombin; DS, degree (number) of sulfation; HAS, hemostasis study; HINT, computerized hydropathic interaction analyses; KD, equilibrium dissociation constant associated with nonionic interactions only; LMWL, sulfated low molecular weight lignin; PBS, pentasaccharide binding site; PT, prothrombin time; SAR, structure–activity relationships; TEG, thromboelastography.

Over the past decade, a clear trend has emerged toward designing large, more highly sulfated structures, which generally exhibit enhanced anticoagulant activity. However, excessive sulfation can paradoxically reduce potency, indicating the need for a balanced degree of functionalization. The molecular structure of these inhibitors also plays a decisive role. For factor XIIIa inhibition, both moderate sulfation and a flexible, linear molecular framework are key determinants of potency [66]. The same structural principles apply to factor XIa inhibitors, where the most active compounds typically combine moderate sulfation, a linear backbone, and a long, flexible linker (see Table 1). Such features enable optimal positioning within allosteric binding regions of serine proteases, thereby maximizing inhibitory efficacy while preserving selectivity.

Recent advances in this field have highlighted the discovery of nonacetylated lignosulfonates (NALS) as promising anticoagulant agents (Table 1). These polymeric structures exhibit a highly selective inhibitory profile toward factor XIa, while showing no significant activity against thrombin or factor Xa. In this context, aromatic polymers demonstrated greater potency than aliphatic analogs and the absence of acetylation within the molecular framework was associated with enhanced selectivity for factor XIa. Moreover, molecular weight was found to be a critical parameter, with medium‐sized polymers providing the optimal balance between activity and selectivity. The salt form also proved essential: sodium salts exhibited pronounced inhibitory activity, whereas potassium salts were inactive. Taken together, the combination of a polyanionic surface, an aromatic backbone, moderate molecular weight, and sodium as the counter‐ion appears to be crucial for achieving selective FXIa inhibition [67]. Accordingly, NALS represents a novel lead scaffold for the development of selective, polymeric FXIa inhibitors, a significant progression beyond previously studied di‐ or trimeric systems, offering both high efficacy and strong enzyme selectivity. As a result, a stronger focus was given on examining polymer structures as heparin mimetics instead of natural compounds for sulfation. Polymeric structures thus appear to be a very interesting and promising approaches, which will be discussed in more detail in the next section.

In summary, the sulfated natural compounds discussed represent an interesting class of anticoagulant agents whose activity has been confirmed in several studies, although their efficacy appears to be limited. In particular, investigations of NALS polymers and their demonstrated potency suggest an emerging trend in the development of heparin mimetics.

### 2.3 Synthetic Polymers as Heparin Mimetics—Sulfated and Non‐Sulfated Polymers

Polymers, particularly highly sulfated non‐carbohydrate structures, are especially well suited as heparin mimetics, as they can simulate both the negative charge density and the molecular size of heparin. Over time, copolymers have gained increasing attention due to their superior anticoagulant properties, which arise from the interplay of their two components and the resulting variations in charge density and functional‐group arrangement.

As early as the 1990s, Silver et al. demonstrated that sulfated polyurethanes (PEUs) can shorten clotting time and reduce thrombus formation in vitro. These effects have been attributed to three main mechanisms: complex formation between thrombin and the polymer, disruption of fibrin polymerization, and interactions of the polymer with plasma antiproteinases and fibrinogen. The solubility and sulfonate content of the polymers are critical in these mechanisms, leading to a heparin‐like effect through without clear dependence on the binding to antithrombin III.

The synthesis of the PEUs has been described in a two‐step approach. First, a PEU backbone was prepared by reacting methylene‐bis(p‐phenyl isocyanate) (MDI), 1,4‐butanediol (BDO), and polytetramethylene oxide (PTMO) in a 3:2:1 molar ratio. This reaction yielded a segmented PEU with urethane groups along the polymer backbone. In the second step, the material was chemically modified by substituting the urethane hydrogens with propyl sulfonate groups. Depending on the degree of substitution, 30%, 40%, or 80% of the urethane nitrogen atoms were functionalized with sulfonate side groups, giving rise to the derivatives PEU‐SO3‐0.30, PEU‐SO3‐0.40, and PEU‐SO3‐0.80 [82].

Sulfated poly(glutamic acid) polymers (Figure 2) also exhibited prolonged clotting times with increasing proportions of sulfate groups, although the significance of this finding was limited to simple clotting tests. The synthesis of poly(γ‐glutamic acid) sulfonate (γ‐PGA sulfonate) was achieved through chemical modification of γ‐PGA. The carboxyl groups of the side chains were linked to the amino group of taurine via a carbodiimide‐mediated coupling, resulting in the formation of amide bonds and the introduction of sulfonate groups into the polymer. The degree of sulfonation could be controlled by varying the reaction conditions. The investigation showed that the anticoagulant effect increases significantly with increasing sulfonation [83].

In addition to the well‐documented anticoagulant effects of highly sulfated polymers, certain non‐sulfated polyanionic polymers also display anticoagulant activity. One example is poly acrylic acid (PAA) (Figure 2). This polymer enhances the inhibition of thrombin by antithrombin by acting as a molecular bridge. As a result, the reaction proceeds at a markedly accelerated rate, in a manner similar to the mechanism of heparin. PAA binds non‐specifically to both the pentasaccharid‐binding site and the heparin‐binding region of antithrombin. Its effect is largely mediated by multivalent electrostatic interactions: the negatively charged carboxylate groups of PAA interact with the positively charged heparin‐binding sites of the protein. Analysis of salt‐dependent dissociation behavior revealed a total of five ionic interactions between PAA and antithrombin. In contrast, non‐ionic interactions were negligible. Compared with heparin, however, these interactions are weaker and less specific. Consequently, higher concentrations of PAA are required to achieve a comparable anticoagulant effect, and the binding affinity is more susceptible to disruption by changes in ionic strength, such as variations in salt concentration. Optimization of the structure would be achieved by optimizing the chain length, charge density, and dosage. In this context, the negative charges of the polymer are primarily localized in a central region. However, more distant negatively charged groups may also interact with additional binding sites beyond the pentasaccharide‐binding region [84]. However, the anticoagulant activity of PAA could not be confirmed in APTT and PT analyses, which will be explained later in this review [85]. PAA is commercially produced either by the hydrolysis of tert‐butyl acrylate or by direct polymerization of acrylic acid (AA) monomers via reversible addition‐fragmentation chain‐transfer (RAFT) polymerization. The RAFT process enables precise control over chain length, targeted end‐group functionality, and low poly dispersity, making it a preferred method for synthesizing these polymers.

The Desai group developed non‐heparin‐like oligomers derived from 4‐hydroxycinnamic acids (caffeic, ferulic, and sinapic acids) as anticoagulants. These compounds inhibit coagulation proteases such as thrombin and FXa through both direct and indirect mechanisms. The synthesized dehydrogenation polymers (DHPs), ranging in size from tetramers to pentadecamers, were tested in both non‐sulfated and sulfated forms. Biological evaluations revealed that both variants exhibited marked anticoagulant activity in coagulation assays. Notably, the sulfated oligomers were two‐ to three‐times more effective than their non‐sulfated counterparts, displaying anticoagulant effects comparable to LMWHs. Mechanistically, these oligomers act directly by inhibiting proteases and indirectly by enhancing or mediating the activity of endogenous inhibitors against thrombin and FXa. This represents one of the first reported examples of a cinnamic acid‐based inhibitor with such a “dual mode of action.” These oligomers were synthesized via a combined chemical and enzymatic approach. In a first step, peroxidase‐catalyzed oxidative coupling converted the 4‐hydroxycinnamic acids into DHPs. The resulting lignin‐like oligomers were then chemically sulfated using a trimethylamine‐sulfur trioxide complex. This chemo‐enzymatic strategy employs mild reaction conditions and generates bond types reminiscent of those found in natural lignin. The use of different cinnamic acid monomers yields distinct oligomer profiles and provides diverse functional groups for further modification. Subsequent sulfation introduces strong negative charges into the molecules, thereby enhancing their anticoagulant activity and rendering them structurally and functionally more similar to heparin [86].

Based on these findings, Henry et al. optimized the DHP structure through sulfation and examined the resulting polymers (CDSO3, FDSO3, and SDSO3) for their effects on the human coagulation factors VIIa, IXa, Xa, and α‐thrombin. Their study assessed both direct and indirect protease inhibition, as well as the reversibility of these effects using protamine neutralization and antithrombin‐binding analyses. The results show that the interaction between the polymers and antithrombin is driven predominantly by non‐ionic binding energies (80%–87%), in clear contrast to the largely ionic binding observed in the heparin‐pentasaccharide‐antithrombin interaction. These polymers, therefore, represent the first examples of antithrombin ligands whose binding is dominated by non‐ionic forces. Furthermore, the data demonstrate that the DHPs interact not only with the pentasaccharide binding site but also with the extended heparin‐binding site of antithrombin [87]. In addition, all three of the compounds investigated selectively inhibit FXa and thrombin by interacting with the anion‐binding exosite II, thereby allosterically disrupting the enzyme's catalytic apparatus [88].

Despite these innovative strategies for developing anticoagulant drugs that target alternative mechanisms, the research focus has shifted from monomeric polymers toward another class of synthetic heparin mimetics.

### 2.4 Synthetic Copolymers as Heparin Mimicking Structural Scaffolds

Over time, copolymers have become increasingly prevalent compared with simple sulfated polymers, with just one monomer. Instead of merely sulfating existing polymers, an increasing number of research groups are focusing on synthesizing copolymers that combine two different monomer structures. This approach enables precise modulation of charge density, functional‐group arrangement, and backbone flexibility. Additional sulfation can then be applied, for example, to fine‐tune the anticoagulant effect of the resulting polymers. This approach brought the resulting materials closer to mimicking the properties of heparin. Notably, the first successful approaches to generate anticoagulant copolymeric structures were directed to surface coating strategies aiming to prevent complications during surgical or related clinical procedures. The research group led by Ren et al. developed polyethersulfone‐based membranes in comparison with heparin‐like macromolecules. The anticoagulant copolymer polystyrene‐co‐acrylic acid (poly(St‐co‐AA)) was the first polymer to contain –COOH, –OH, and –SO3H groups, and it was used as an additive for conventional biomaterials, whereby it should be noted that sulfonation was carried out after polymerization. This copolymer was synthesized via reversible addition‐fragmentation chain‐transfer (RAFT) polymerization. The copolymerization of styrene and AA was carried out in the presence of azobisisobutyronitrile (AIBN) and tert‐butanol (t‐BuOH), using a carboxyl‐terminated trithiocarbonate as RAFT agent under a heated, nitrogen‐rich atmosphere. RAFT polymerization was employed for the first time to synthesize well‐defined heparin‐like polymers containing multiple functional groups. In vitro experiments demonstrated that the polymer‐modified coatings exhibited superior anticoagulant properties compared with the unmodified ones. This enhancement was attributed to the presence of –SO3H, –COOH, and –OH groups, whose anionic and polar characteristics enable the binding of coagulation factors, thereby reducing coagulation activity [89].

Building on this prior knowledge, the same research group developed additional anticoagulant copolymers that demonstrated good anticoagulant performance. These polymers were collectively classified as negatively charged macromolecules (NCMs). Using a phase inversion technique, the NCMs were incorporated into polyethersulfonate (PES) membranes, allowing the functional groups responsible for anticoagulant activity to become enriched at the membrane surface. The modified membranes exhibited markedly enhanced anticoagulant properties, as confirmed by measurements of the activated partial thromboplastin time (APTT). Among the tested polymers, poly (acrylic acid‐co‐methyl methacrylate) (poly(AA‐co‐MMA)), combined anticoagulant activity with high compatibility within the membrane. As in their previous study, the multi‐step synthesis began with RAFT polymerization to produce the polymer backbone using specific chain transfer agents. Subsequent sulfation was performed with concentrated sulfuric acid at 3:1 mass ratio relative to the copolymer. This process introduced negatively charged –SO3H groups into the macromolecular structure, further enhancing the anticoagulant effect [90].

In the ongoing development of the anticoagulant biomembranes, heparin‐like poly(ether sulfone) polymers (HLPES) were introduced to replicate the anticoagulant efficacy of heparin without relying on the heparin molecule itself, while maintaining the well‐established advantages of poly(ether sulfone) (PES) as a biomembrane material. Incorporating the heparin‐like component NNPES into the polymer, thus forming HLPES, significantly reduced protein adsorption compared with the conventional PES membrane. Moreover, both APTT and TT measurements demonstrate prolonged coagulation times in the presence of the modification, indicating enhanced blood compatibility. Additional analyses also revealed reduced platelet adhesion and activation on the modified membranes, further supporting the improved hemocompatibility of HLPES [91].

**Scheme 1:**
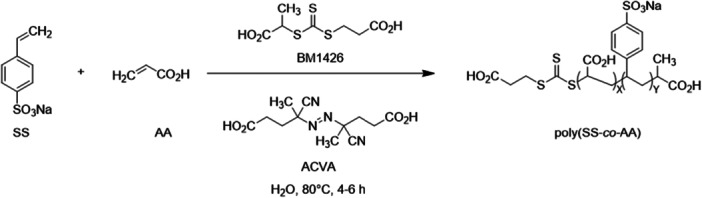
RAFT polymerization of sodium 4‐styrenesulfonate copolymers (SS). Abbreviations: acrylic acid (AA); ((((1‐carboxyethyl)thio)carbonothioyl)thio)propanoic acid (BM1429); 4,4′‐azobis(4‐cyanovaleric acid) (ACVA).

**Figure 3:**
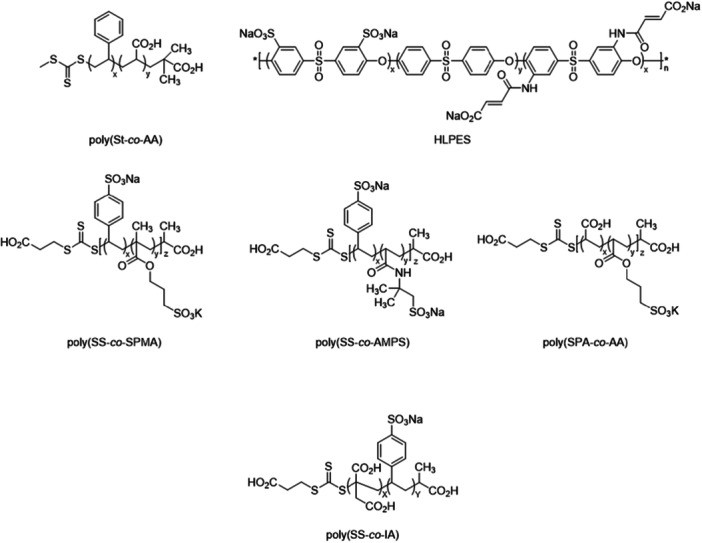
Fully synthetic copolymers with anticoagulant effect.

The Ferro group focused on developing a library of heparin mimetics derived from homo‐ and copolymers, with particular emphasis on sulfonated monomers. They synthesized and characterized homopolymers, prepared in various compositions and molecular weights, including sodium 4‐styrene sulfonate (SS), potassium 3‐sulfopropyl acrylate (SPA), potassium 3‐sulfopropyl methacrylate (SPMA), sodium 2‐acrylamido‐2‐methyl‐1‐propane sulfonate (AMPS), and AA. Furthermore, they generated copolymers in different combinations and ratios of the indicated sulfonated monomers with non‐sulfonated species, such as AA, or as combinations of two of these sulfonated monomers and subsequently evaluated based on polymer size. Their anticoagulant activity was assessed using APTT and thrombin clotting time (TCT) assays. By systematically varying polymer compositions and molecular mass, the group aimed to gain deeper insights into SARs and thereby optimize the design of synthetic heparin mimetics. In APTT assays, the homopolymers exhibited stronger anticoagulant activity than the corresponding copolymers. Moreover, higher molecular weight was associated with enhanced anticoagulant efficacy. By combining different monomers, copolymers with distinct affinities to target proteins were obtained. The copolymers were of particular interest for investigating SARs. However, the homopolymers poly(sodium 4‐styrene sulfonate) [poly(SS)] exhibited the strongest anticoagulant activity in the presented in vitro assays. Copolymers containing this monomer also demonstrated greater anticoagulant potency compared with other compositions. The copolymers were synthesized via RAFT polymerization, as previously described for the other polymers, using ((((1‐carboxyethyl)thio)carbonothioyl)thio)propanoic acid (BM1429) as the thermal initiator under microwave irradiation (Scheme 1) [85]. At the same time, they synthesized additional polymers containing both carboxylated and sulfated monomers (Figure 3). In addition to the previously mentioned APTT and TCT assays, further analyses were conducted, including thrombin generation tests, anti‐FXa and IIa activity measurements, as well as cytotoxicity evaluations of the polymers. A particularly important observation was made regarding the introduced monomer poly(acrylic acid) [poly(AA)]. Poly(AA) showed no efficacy in the APTT and TCT coagulation assays. However, when combined with poly(SS) (referred to as poly(SSS) in this publication) to form a copolymer, it showed pronounced anticoagulant properties comparable to those of UFH and LMWH in the APTT assay and to LMWH in the TCT assay. The copolymer poly(SSS‐co‐AA) 1:1 20 kDa proved to be particularly effective. Moreover, in subsequent anti‐FIIa assays, this copolymer was the only one to show an AT‐independent inhibitory effect on thrombin. It thus represents one of the first synthetic copolymers known to exhibit this activity. The underlying reason for this may lie in the specific arrangement and chemical nature of the sulfated and carboxylated groups within this molecule, which favor interactions with thrombin. Although significantly higher polymer concentrations were required to achieve comparable effects in the in vitro assays, no notable cytotoxicity was observed. They thus demonstrated that sulfonated homopolymers provide a favorable balance between efficacy and biocompatibility and that target copolymerization with AA can not only enhance anticoagulant activity but also additional functionalities, such as FIIa inhibition, into the polymers functional profile [92]. In extended biological studies, the polymers from both investigations were evaluated for their anticoagulant activity, their effects on platelets, and their interactions with selectins and other proteins relevant to metastatic processes. In addition, the ability of these polymers to inhibit melanoma cell adhesion to endothelial cells under dynamic blood flow conditions was assessed. Among the tested materials, poly(SSS) and poly(SSS‐co‐AA) 1:1 at 10 kDa and 20 kDa exhibited comparable or superior performance to the reference compounds UFH and LMWH. The size and composition of the polymers proved to be critical determinants of their biological activity. Polymers with very low or very high molecular weights (approximately 5 kDa and 50 kDa) displayed weak or negligible effects, whereas those in the 10–20 kDa range demonstrated the highest efficacy [93].

This polymer library was expanded by introducing copolymers containing IA as a carrier of additional carboxyl groups (Scheme 1/ Figure 3). The incorporation of itaconic acid contributed an extra carboxyl functionality to the polymer backbone, enhancing its anionic character. Various copolymer compositions were synthesized by combining itaconic acid with the previously mentioned sulfonated monomers SS, AMPS, and SPMA while systematically varying both the monomer ratios and the polymer chain lengths. A broader range of anticoagulant bioassays was performed using these copolymers. Among them, poly(SS‐co‐IA) copolymers with a 1:1 monomer ratio and molecular weights between 10 and 20 kDa demonstrated the most consistent and potent biological activity. Importantly, these polymers exhibited low cytotoxicity at effective concentrations, indicating minimal interference with cell proliferation. Beyond their anticoagulant properties, the poly(SS‐co‐IA) copolymers showed a more than 1000‐fold lower binding affinity to PF4 compared with UFH, suggesting a reduced risk of heparin‐induced complications. Interestingly, another polymer, poly(SPMA‐co‐IA), displayed superior efficacy in inhibiting heparanase activity. This finding underscores that varying the monomer composition within these copolymers can selectively modulate their mechanisms of action and molecular targets. These polymers were synthesized using RAFT polymerization, similar to the procedure previously used [94].

### 2.5 Fully Synthetic Heparin Mimetic Polymers—Therapeutic Perspectives Beyond Anticoagulation

Given the pleiotropic effects of heparin, a key question concerning heparin mimetics is whether, in light of their comparable anticoagulant efficacy, they also modulate similar molecular targets and whether these targeted structural modifications can be enhanced or diminished by structural fine tuning. Already in the early 2000s, several research groups began exploring whether their synthesized polymers possessed additional biological properties beyond anticoagulation. Matsusaki et al. investigated the effects of their anticoagulant polymer poly(γ‐glutamic acid)‐sulfonate on fibroblast growth factor 2 (FGF2), which plays a key role in physiological processes such as cell proliferation, angiogenesis, wound healing, and tissue regeneration. Their results demonstrated that the polymer had high FGF2 activity to a degree comparable to that of heparin [95].

To assess the efficacy in a malignant, metastatic context, fully synthetic heparin‐mimetic polymers of the Ferro group were evaluated for their ability to inhibit heparanase. Heparanase is regarded as a marker of poor tumor prognosis because its cleavage of heparan sulfate chains on mammalian cell surfaces can promote tumor growth, drive angiogenesis, and facilitate metastasis. The polymers investigated, poly(SSS) 20 kDa, poly(SSS‐co‐AA) 1:1 (10 and 20 kDa), and poly(SPA‐co‐AA) 1:1 20 kDa, exhibited strong inhibition of the enzyme, surpassing even the potency of the clinically tested heparanase inhibitor pixatimod (PG545). Furthermore, binding‐affinity analyses and flow‐chamber assays demonstrated interactions with VLA‐4 and P‐selectin, revealing reduced adhesion of MV3 melanoma cells to endothelial cells in the presence of these polymers, particularly poly(SSS‐co‐AA) 1:1 20 kDa. Collectively, these findings highlight potential points of therapeutic intervention in the metastatic cascade, as demonstrated in the in vitro experiments [93].

In the wake of the heightened interest in heparin during the coronavirus pandemic, synthetic polymers have likewise emerged as a compelling area of research. Studies on SARS‐CoV‐2 have shown that the copolymer poly(SS‐co‐AA) 1:1 20 kDa, referred to as HMSA‐06‐20 in the publication, exhibits pronounced antiviral activity. The data demonstrated that HMSA‐06 can inhibit both viral cell entry and viral replication. These effects were analyzed in vitro using three different cell lines: Vero E6 and Calu3 for the SARS‐CoV‐2 prototype and additionally Caco2 cells for the Omicron variant. The antiviral potency of the polymer appeared to be influenced, among other factors, by its molecular weight. While the 20 kDa variant HMSA‐06‐20 showed strong inhibitory effects, the smaller 5 kDa version (HMSA‐06‐05) was noticeably less effective. Under similar conditions, heparin also displayed substantially weaker inhibition of SARS‐CoV‐2 cell entry compared with the synthetic polymer. Beyond its antiviral function, HSMA‐06 also suppressed NLRP3‐mediated inflammatory responses. In parallel with these antiviral studies, the effects of the mimetic on cellular autophagy were investigated. Here as well, HMSA‐06‐20 induced the strongest increase in the autophagy marker LC3II. These results correlated with SARS‐CoV‐2 S‐ and N‐protein expression after infection: the higher autophagy was upregulated, the more pronounced the decline in protein expression was [96].

In addition to SARS‐CoV‐2, Nahain et al. investigated the efficacy of heparin mimetic polymers against several mosquito‐borne viruses, including dengue virus (DENV), Zika virus (ZIKV), Ross River virus (RRV), and Yellow fever virus (YFV). In this study, the homopolymer poly(SS) emerged as a broad‐spectrum antiviral agent with particularly strong activity. Notably, its pronounced efficacy against YFV and ZIKV was demonstrated in both mosquito‐derived and human target cell lines. It is important to note here that the concentration of poly(SS) required to inhibit viral infection are substantially lower than those needed to elicit anticoagulant effects. This indicates that the antiviral and anticoagulant properties of the polymer can be modulated independently through concentration adjustment. Copolymers of SS with other functional groups also displayed antiviral activity, although this activity directly correlated with the presence of SS [97].

In subsequent studies, these polymers were evaluated for their immunomodulatory properties. The polymers poly(SSS) 20 kDa, poly(SSS‐co‐AA) 1:1 (10 and 20 kDa) and poly(SPA‐co‐AA) 1:1 20 kDa showed a significant ability to downregulate the differentiation of naïve CD4+ T cells into regulatory T cells, indicating a potential immune stimulatory effect. Although these effects could only be partially reproduced in other immune cell types, such as CD8+ T cells and natural killer cells, the polymers nevertheless exhibited modulatory activity that could likely be enhanced through further structural optimization. These findings raise intriguing questions regarding the precise mechanisms by which the polymers influence immune responses and which cytokines might serve as key targets for modulation [98].

In addition to AA copolymers, the indicated library of itaconic acid (IA) containing copolymers displayed activities beyond their anticoagulant properties. The polymers poly(SPMA‐co‐IA) 2:1 and poly(SPMA‐co‐IA) 4:1 (5–20 kDa) demonstrated comparable, and in some cases slightly superior, heparanase inhibition relative to the clinically tested candidate PG545. Notably, despite their strong heparanase‐inhibitory activity, these polymers exhibited weaker anticoagulant effects than the other candidates examined [94].

Based on these results, it can be concluded that polymeric heparin mimetics, like heparin itself, exhibit pleiotropic effects. Moreover, variations in charge density, molecular size, and the composition of functional groups allow SARs to be more systematically investigated and tailored to the intended application.

## 3 Conclusion and Future Outlook

In summary, the studies discussed above indicate that sulfated structures featuring a flexible backbone and intermediate molecular weight exhibit the strongest anticoagulant activity. However, it is not only the amount of sulfation that appears to be decisive but also the arrangement in the molecule relative to other functional groups. Recent investigations highlight polymers as particularly promising candidates, as their tunable composition, often comprising one or more distinct monomers, allows them to closely mimic the anticoagulant efficacy of heparin while providing structurally more robust and reproducible alternatives.

When comparing the two design strategies for heparin mimetics, sulfated natural compounds provide an elegant approach for achieving the desired level of anticoagulant activity and target interaction with only minor structural modifications. In contrast, polymeric mimetics exhibit a broader range of potencies and offer applications that extend beyond anticoagulation. Despite these advantages, polymer‐based systems face the challenge of structural heterogeneity, which represents a significant limitation in polymer drug synthesis. This issue is further compounded by the difficulties of precisely controlling the polymerization process. Although modern synthetic techniques, such as RAFT polymerization, have improved control over polymer architecture, precise regulation remains limited. Consequently, strict in‐process controls and standardized manufacturing procedures are required to minimize these risks.

Beyond anticoagulation, several of these synthetic polymeric structures display activity against additional biological targets, and their potency can be modulated through targeted structural modifications. This demonstrates that the pleiotropic effects traditionally attributed to heparin can not only be reproduced but potentially enhanced in synthetic analogs through systematic SAR studies. It should be noted, however, that the majority of these findings are based on in vitro assays, including binding studies, and that the true extent of their biological efficacy must be assessed in in vivo models and ultimately clinical investigations to enable definitive conclusions.

For example, key aspects such as the pharmacokinetics and pharmacodynamics in the human body, as well as the metabolism and safety profiles of individual compounds, have not yet been evaluated in clinical trials. Although these substances and the corresponding analyses provide promising prospects for potential drug candidates, they do not yet offer sufficient insight into their translational applicability.

Nevertheless, the structures investigated provide valuable insight into the multifaceted activity of heparin, both in its interactions with individual proteins and with the coagulation cascade as a whole. They also enable the rational design of compounds tailored to specific biological targets, thereby supporting the development of more selective therapeutic strategies. Consequently, heparin, despite its nearly century‐long history in clinical use and commercial production, should not be regarded as an outdated drug. Instead, current studies continue to highlight its non‐anticoagulant activities and underscore its relevance as a lead structure for the development of new synthetic analogs.

## Conflicts of Interest

The authors declare no conflicts of interest.
